# Deciphering the Impact of *EPHA1‐AS1* Gene Polymorphism on Social Cognition Deficits in Parkinson's Disease

**DOI:** 10.1002/cns.70801

**Published:** 2026-03-27

**Authors:** Yu‐Chen Lin, Chun‐Hsiang Tan, Wei‐Pin Hong, Rwei‐Ling Yu

**Affiliations:** ^1^ School of Medicine, College of Medicine National Cheng Kung University Tainan Taiwan; ^2^ Department of Neurology, Kaohsiung Medical University Hospital Kaohsiung Medical University Kaohsiung Taiwan; ^3^ Graduate Institute of Clinical Medicine, College of Medicine Kaohsiung Medical University Kaohsiung Taiwan; ^4^ Department of Neurology, National Cheng Kung University Hospital, College of Medicine, National Cheng Kung University Tainan Taiwan; ^5^ Institute of Behavioral Medicine, College of Medicine National Cheng Kung University Tainan Taiwan; ^6^ Institute of Allied Health Sciences, College of Medicine National Cheng Kung University Tainan Taiwan; ^7^ Department of Psychiatry, National Cheng Kung University Hospital, College of Medicine National Cheng Kung University Tainan Taiwan; ^8^ Office of Sustainability and Strategic Development National Cheng Kung University Tainan Taiwan

**Keywords:** *EPHA1‐AS1* gene, healthcare, inflammatory, Parkinson's disease, Reading the Mind in the Eyes Test, social cognition

## Abstract

**Aims:**

Ephrin type‐A receptor 1 (EPHA1) has been identified as a potential contributor to the pathogenesis of Parkinson's disease (PD). The complex interactions between PD symptoms and the EPHA1 protein warrant further exploration.

**Methods:**

A cohort of 509 participants, including 362 normal controls (NCs) and 147 PD patients, was included and genotyped for the ephrin type‐A receptor 1‐antisense 1 (*EPHA1‐AS1*) gene. Overall and emotion‐specific social cognition were assessed using the Reading the Mind in the Eyes Test. The relationship between the *EPHA1‐AS1* gene, PD, and social cognition was analyzed using the Mann–Whitney U test, the Quade test, and moderation analysis.

**Results:**

Compared to NCs, PD patients showed markedly poorer social cognition, especially in overall and negative emotions, regardless of genotype. The rs2966700 variant significantly affects the positive subscore among all participants (*p* = 0.006), with TT carriers performing better. Furthermore, PD was a significant moderator of the association between rs2966700 and the positive subscore (*p* = 0.001), with PD patients carrying the C allele performing worse.

**Conclusions:**

This study offers new insights into the interaction between the *EPHA1‐AS1* gene and PD, thereby enhancing our understanding of its impact on social cognition, particularly in recognizing positive emotions in individuals with PD.

## Graphical abstract

Patients with Parkinson's disease showed poorer social cognition overall regardless of gene. There is an interplay between PD, rs2966700, and positive emotion recognition. PD patients carrying the C allele on rs2966700 performed poorer, suspected due to decreased *EPHA1‐AS1* activity and increased inflammation.

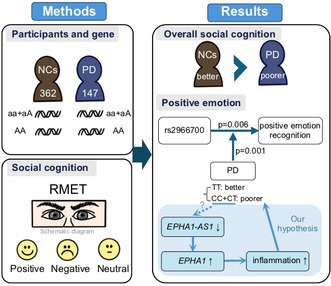

## 1 Introduction

### 1.1 Social Cognition and Its Assessment

In the intricate tapestry of human neural functions, social cognition is pivotal for interpreting and responding to the myriad social stimuli encountered daily. It encompasses the processes involved in perceiving, encoding, storing, retrieving, and regulating information about oneself and others, thereby supporting effective interpersonal interactions [1]. Key components include emotion recognition and theory of mind (ToM), which is further bifurcated into cognitive and affective ToM [2, 3, 4]. The neural substrates underpinning social cognition are distributed across various brain regions. Emotion recognition primarily involves the ventral system, including the amygdala, insula, ventral striatum, anterior cingulate gyrus, and prefrontal cortex [3], whereas ToM involves the orbital frontal striatal circuit and the dorsolateral frontal striatal loop [5]. These neural circuits collectively orchestrate our ability for social interactions.

One prominent tool for evaluating social cognition is the Reading the Mind in the Eyes Test (RMET), developed by Baron‐Cohen and colleagues [6]. The RMET assesses both emotion recognition and ToM [3] through images featuring eyes and it encompasses a diverse range of emotions, including positive, negative, and neutral emotions. Prior studies have segmented the RMET's 36 questions based on emotion valence, a method we adopted following a 2005 study by Harkness et al. [7] This categorization has been supported by subsequent research [8, 9].

The importance of social cognition is underscored by its significant influence on daily interactions, which has led to increased research into its determinants [8, 10, 11]. A 2022 study by Lee et al. demonstrated that enhanced social cognition correlates with younger age, female gender, higher educational attainment, improved cognitive function, and fewer depressive symptoms [10]. Additionally, damage to specific brain regions and the depletion of certain neurotransmitters, such as dopamine, can impair social cognition [12]. Parkinson's Disease (PD), characterized by the loss of dopamine, is a prime example of a condition that affects social cognitive abilities [13, 14].

### 1.2 The Impact of Parkinson's Disease on Social Cognition

PD is defined by the degeneration of dopaminergic neurons, leading to both motor and nonmotor symptoms [15, 16, 17, 18]. Studies suggest that biological, psychological, and social factors [19, 20, 21, 22] can influence these non‐motor symptoms in individuals with PD, potentially impacting their quality of life [23] and social functionality [2, 24]. Recently, the study of social cognitive functions has emerged as a prominent area of research [25, 26]. Compared to normal controls (NCs), PD patients exhibit diminished social cognition [26, 27] and often struggle more with negative emotions [26, 28, 29]. These findings highlight the relevance of social cognition in PD and its potential for early symptom identification.

### 1.3 EPHA1‐AS1 and EPHA1

Ephrin type‐A receptor 1‐antisense 1 (*EPHA1‐AS1*) is a sequence complementary to the ephrin type‐A receptor 1 (*EPHA1*) mRNA, which may regulate *EPHA1* expression through binding and translational inhibition [30]. This interaction is thought to affect EPHA1 protein levels and activity. Ephrin receptors are critical tyrosine kinases involved in various diseases and are divided into the ephrin A and ephrin B groups, each playing unique roles in cellular signaling [31]. The binding of these receptors to their respective ligands is crucial for brain endothelial cell contraction, T cell recruitment, axonal guidance, and synaptic plasticity [31, 32]. Additionally, EPHA1 has been implicated in the proinflammatory role in tumor development through angiogenesis [33], acting as a ligand for epidermal growth factor receptor [34], and positively correlated with interleukin‐6 and vascular endothelial growth factor levels [35].

On the other hand, EPHA1 has been linked to neurodegenerative diseases such as Alzheimer's Disease (AD) and PD [33, 36, 37, 38]. In PD, increased EPHA1 expression has been documented in PD mouse brain tissue, in the peripheral blood of PD patients, and in a PD cell model derived from SH‐SY5Y cells. Overexpression of EPHA1 resulted in alpha‐synuclein accumulation and activation of the C‐X‐C motif chemokine ligand 12/C‐X‐C chemokine receptor type 4 (CXCL12/CXCR4) pathway, linking it to PD pathogenesis and neuroinflammation, particularly in promoting astrocyte activity [39, 40].

Regarding its genetic aspects, *EPHA1* has been identified as a risk gene for AD, associated with inflammation and specific genetic polymorphisms [36, 38]. The rs20217856 single‐nucleotide polymorphism (SNP) in *EPHA1* is linked to late‐onset AD and reduced reverse signaling capacity, potentially impacting the blood–brain barrier (BBB) [41]. In contrast, SNPs rs11767557 and rs11771145 have been associated with a decreased risk of late‐onset AD [42].

Despite increasing support for EPHA1 in neuroinflammation and PD pathogenesis, the antisense transcript, *EPHA1‐AS1*, remains uncharacterized, particularly in neurodegenerative contexts. *EPHA1‐AS1* has been found to be upregulated in psoriatic skin, an inflammatory disease, in a study analyzing the transcripts of long noncoding RNA [43]. To date, no studies have directly explored the function of *EPHA1‐AS1* or its potential impact on PD or cognitive domains such as social cognition, a critical yet often overlooked non‐motor symptom of PD that typically emerges early and significantly affects quality of life. This gap underscores the need to elucidate the role of *EPHA1‐AS1* in PD.

### 1.4 Study Aim

Our study aims to explore the relationship between PD and EPHA1 specifically through the lens of the *EPHA1‐AS1* gene, thereby enhancing our understanding of EPHA1 protein function. Given the established link between EPHA1 and PD‐related neuroinflammation, this disease provides a critical context for advancing our understanding and exploring deeper aspects of PD pathology. Social cognition was selected as the core clinical variable due to its early and profound impact on patients' interpersonal functioning and daily life [29, 44]. By emphasizing this aspect, we aim to generate insights that aid early detection and strategies for managing PD's non‐motor symptoms.

## 2 Methods

### 2.1 Assessments

#### 2.1.1 Demographic and Clinical Characteristics

We collected demographic and clinical data of all participants through interviews and medical records. The Hoehn and Yahr stages were evaluated using the Movement Disorder Society‐sponsored Revision of the Unified Parkinson's Disease Rating Scale (MDS‐UPDRS) [18]. Cognitive function was evaluated using the Mini‐Mental State Examination (MMSE) [45] for all participants.

#### 2.1.2 Social Cognition Evaluation

Social cognition was evaluated using the RMET [6]. The RMET consists of 36 questions, each displaying only the eye region of a face alongside four descriptive mental or emotional states. Participants selected the descriptor that best matched the depicted emotion. This test includes eight positive, 12 negative, and 16 neutral emotions [7]. Scoring was categorized into positive, negative, and neutral subscores based on specific questions aligned with each emotional tone. Questions 1, 6, 16, 20, 21, 25, 30, and 31 were categorized as positive subscores. Questions 2, 5, 11, 14, 17, 22, 23, 26, 27, 34, 35, and 36 were categorized as negative subscores. Questions 3, 4, 7, 8, 9, 10, 12, 13, 15, 18, 19, 24, 28, 29, 32, and 33 were categorized as neutral subscores.

### 2.2 Participants

This study included 509 Taiwanese participants, including 362 NCs and 147 patients with PD. The NCs group comprised 97 males and 265 females, with ages ranging from 18 to 92 years (mean = 59.13, standard deviation, SD = 16.88). Patients with PD, including 95 males and 52 females, aged 42–85 years (mean = 65.48, SD = 8.56), were diagnosed at National Cheng Kung University Hospital and Kaohsiung Medical University Hospital. Participants were recruited from surrounding regions and required basic reading skills. Exclusion criteria included a history of brain surgery, stroke, brain injury, dementia, or schizophrenia. All participants provided consent before the examinations. All study procedures were approved by the Ethics Research Committee of National Cheng Kung University Hospital (approval number: A‐ER‐109‐491), and all methods were performed in accordance with the approved guidelines.

### 2.3 Genotyping

Venous blood samples were collected for DNA extraction in hospitals or designated inspection centers, following standard protocols. We genotyped 35 SNPs using the Axiom Genome‐Wide TWB 2.0 Array Plate, developed by Thermo Fisher Scientific for Taiwanese populations. Only 18 SNPs with a minor allele frequency above 10% were included in the linkage disequilibrium analysis, and detailed data were listed in the Table S1. Subsequently, using Haploview version 4.2 [46] and the definition of “solid spine of LD” for the analysis, seven linkage disequilibrium blocks were generated from the 18 SNPs. The SNP with the highest minor allele frequency in each block was selected for analysis in this study. Therefore, a total of 7 SNPs were included in the analysis of this study, including rs12703526, rs11771145, rs7805776, rs9640385, rs9640386, rs2966700, and rs2949770. All SNPs conformed to the Hardy–Weinberg equilibrium. The number of participants by genotype is shown in Table S2.

### 2.4 Statistical Analysis

Statistical procedures were performed using IBM SPSS Statistics version 31. We assessed the Hardy–Weinberg equilibrium for all SNPs. Nonparametric analysis methods are employed in the further analyses due to the non‐normally distributed data, as indicated by the Kolmogorov–Smirnov test. Spearman's correlation was used to analyze the relationships among Hoehn‐Yahr stage, levodopa equivalent daily dose (LEDD), MMSE score, and RMET scores. Demographic characteristics between the PD and NC groups were compared using the chi‐square test for categorical data and the Mann–Whitney *U* test for continuous data.

We divided the PD and NC groups into two subgroups based on whether they carried the minor allele or not. To analyze differences in social cognition between PD and NCs, we compared PD and NCs using RMET scores as dependent variables. To test the hypothesis that genotype might affect social cognition, we compared the RMET score of two subgroups (with and without the minor allele) within PD and within NCs. All statistical analyses were conducted using the Mann–Whitney U test. Furthermore, to avoid possible factors that might overshadow the effect of genes on social cognition, we analyzed sex, age, education level, and MMSE score as covariates using the Quade test. For comparison within the PD group, we additionally included Hoehn‐Yahr stage and LEDD as covariates for the Quade test. The statistical power was calculated by a post hoc power analysis with G*Power 3.1 [47] based on an ANCOVA model as an approximation of the nonparametric Quade test. A medium effect size (*f* = 0.25) and alpha value of 0.0125 due to Bonferroni correction were set.

To explore whether having PD or not affects the effect of the gene, we conducted a moderation regression analysis using Model 1 from the SPSS macro Process 4.2 [48]. The independent variable was genotype, the dependent variable was the RMET score, and the moderator was whether the participant had PD. The analysis was conducted with sex, age, education level, and MMSE score as covariates. We used the Bonferroni correction to minimize the risk of type I error arising from multiple comparisons. Since we conducted four types of independent variables in all statistical processes, including the RMET total score, positive subscore, negative subscore, and neutral subscore, we adjusted the alpha value to 0.0125 (0.05 divided by 4). Therefore, *p* values less than 0.0125 were considered significant in our study.

### 2.5 Language Editing

ChatGPT (OpenAI, GPT‐4 model) was used for English grammar correction. All content was carefully reviewed and approved by the authors to ensure accuracy and integrity.

## 3 Results

### 3.1 Demographic and Clinical Characteristics

A total of 509 participants were included in this study, including 362 NCs and 147 patients with PD. The detailed number of demographic and clinical characteristics are shown in Table 1. Regarding demographic characteristics, the PD group has a higher percentage of male participants and a higher mean age. Additionally, a negative correlation between Hoehn‐Yahr stage and the RMET positive subscore (*r* = −0.216, *p* = 0.009) was observed. There was also a positive correlation between MMSE score and RMET total score (*r* = 0.297, *p* = < 0.001), positive subscore (*r* = 0.131, *p* = 0.003), negative subscore (*r* = 0.221, *p* = < 0.001), and neutral subscore (*r* = 0.235, *p* = < 0.001). As for overall cognition and negative RMET subscore, the NCs performs better than the PD. However, the RMET positive and neutral subscores showed no significant difference after controlling for covariates between PD and NCs.

**TABLE 1:** Demographic and clinical characteristics of NCs and PD group.

	NCs		PD		Stat.	*p*
	Mean	SD	Mean	SD		
Sample size	362	—	147	—	—	—
Gender, Male (%)	97 (26.80%)	—	95 (64.63%)	—	63.689^a^	< 0.001*
Age (years)	59.13	16.88	65.48	8.56	22761.5^b^	0.011*
Education (years)	12.88	3.45	12.30	3.78	28755.5^b^	0.144
MMSE	27.60	2.46	26.43	2.51	35342.5^b^	< 0.001*
RMET‐total score	20.60	4.62	18.14	4.25	9.544^c^	0.002*
RMET‐positive subscore	3.72	1.67	3.24	1.59	0.479^c^	0.489
RMET‐negative subscore	6.93	2.22	5.78	2.01	10.316^c^	0.001*
RMET‐neutral subscore	10.17	2.42	9.12	2.46	6.054^c^	0.014
Onset age (years)	—	—	59.06	9.57	—	—
Disease duration (years)	—	—	6.09	5.05	—	—
Hoehn‐Yahr stage	—	—	1.83	0.84	—	—
LEDD	—	—	572.59	659.82	—	—

Abbreviations: LEDD, levodopa equivalent daily dose; MMSE, Mini‐Mental Stage Examination; NCs, normal controls; PD, Parkinson's disease; RMET, Reading the Mind in the Eyes Test; SD, standard deviation; Stat., statistical value.

^a^ Chi‐squared test.

^b^ Two‐tailed independent *t*‐test.

^c^ Quade test (sex, age, education, and Mini‐Mental State Examination score as covariates).

* *p* < 0.0125.

### 3.2 Comparison Between NCs and PD, Within NCs and Within PD

Within‐group comparisons refer to minor allele carrier versus noncarrier comparisons within NCs or within PD. Between‐group comparisons refer to PD versus NCs comparisons within the same genotype stratum. Sex, age, education, MMSE score, and additional Hoehn‐Yahr stage and LEDD for the PD group are controlled as covariates in the Quade test for within‐group and between‐group comparisons.

#### 3.2.1 RMET Total Score

The mean total score and standard deviation for each subgroup of the 7 selected SNPs are displayed in Table 2. The number of participants in subgroups of the 7 SNPs is shown in Table S2. For all subgroup comparisons within NCs and within PD, there is no significant difference using the Mann–Whitney *U* test. After controlling for covariates, subgroup comparisons remain nonsignificantly different within NCs and within PD.

**TABLE 2:** Comparison of RMET total score between and within groups of 7 selected SNPs.

	NCs		PD		Stat.^a^	*p* ^a^	Stat.^b^	*p* ^b^
	Mean	SD	Mean	SD				
rs12703526								
TT+TG	20.340	5.493	17.490	4.052	3.827	< 0.001*	2.590	0.110
GG	20.707	4.238	18.459	4.334	4.306	< 0.001*	6.019	0.015
Stat.^c^	0.028		1.307					
*p* value^c^	0.977		0.191					
Stat.^d^	0.126		0.721					
*p* value^d^	0.723		0.397					
rs11771145								
AA+AG**	20.628	4.611	18.505	4.140	4.546	< 0.001*	6.921	0.009*
GG	20.527	4.687	17.000	4.453	3.707	< 0.001*	2.583	0.110
Stat.^c^	−0.130		−1.521					
*p* value^c^	0.897		0.128					
Stat.^d^	0.014		2.202					
*p* value^d^	0.906		0.140					
rs7805776								
AA+AG	20.486	4.771	18.516	4.202	3.789	< 0.001*	3.854	0.051
GG**	20.770	4.416	17.442	4.300	4.581	< 0.001*	6.428	0.012*
Stat.^c^	0.625		−1.280					
*p* value^c^	0.532		0.200					
Stat.^d^	0.508		1.439					
*p* value^d^	0.476		0.232					
rs9640385								
TT+TC**	20.943	5.015	17.968	4.436	4.433	< 0.001*	6.745	0.010*
CC	20.393	4.350	18.262	4.134	3.904	< 0.001*	3.144	0.077
Stat.^c^	−1.474		0.593					
*p* value^c^	0.140		0.553					
Stat.^d^	0.620		0.763					
*p* value^d^	0.431		0.384					
rs9640386								
AA+AG**	21.000	4.227	18.014	3.758	4.966	< 0.001*	9.638	0.002*
GG	20.158	4.909	18.257	4.714	3.171	0.002*	1.078	0.300
Stat.^c^	−1.483		0.408					
*p* value^c^	0.138		0.683					
Stat.^d^	1.880		0.159					
*p* value^d^	0.171		0.690					
rs2966700								
CC+CT**	20.898	4.525	17.838	4.132	5.889	< 0.001*	15.213	< 0.001*
TT	20.170	4.749	18.750	4.475	1.809	0.070	0.034	0.854
Stat.^c^	−1.553		1.589					
*p* value^c^	0.120		0.112					
Stat.^d^	2.210		3.420					
*p* value^d^	0.138		0.066					
rs2949770								
CC+CA	20.573	4.515	18.424	4.472	2.418	0.016	1.114	0.293
AA**	20.614	4.676	18.053	4.205	5.314	< 0.001*	8.808	0.003*
Stat.^c^	0.160		−0.505					
*p* value^c^	0.873		0.613					
Stat.^d^	0.009		0.148					
*p* value^d^	0.926		0.701					

*Note:* The two subgroups are divided by carrying minor allele or not, shown as minor/minor+minor/major subgroup and major/major subgroup in this table (e.g., TT+TG subgroup and GG subgroup in rs12703526). The number of participants in different genotype subgroups are listed in Table S2.

Abbreviations: NCs, normal controls; PD, Parkinson's disease; RMET, Reading the Mind in the Eyes Test; SD, standard deviation; SNP, single nucleotide polymorphism; Stat., statistical value.

^a^ Comparison between NCs and PD who carry the same genotype using Mann–Whitney *U* test.

^b^ Comparison between NCs and PD who carry the same genotype using Quade test (sex, age, education, and Mini‐Mental State Examination score as covariates).

^c^ Comparison of two subgroups (with and without carrying minor allele) within NCs and within PD using Mann–Whitney *U* test.

^d^ Comparison of two subgroups (with and without carrying minor allele) within NCs and within PD using Quade test (sex, age, education, and Mini‐Mental State Examination score as covariates for NCs group; sex, age, education, Hoehn‐Yahr stage, levodopa equilivant daily, and Mini‐Mental State Examination score dose as covariates for PD group).

* *p* < 0.0125.

** Genotypes that showed a significant RMET difference between NCs and PD after controlling sex, age, education level, and Mini‐Mental State Examination score.

For the between‐group comparison, significant differences are observed in Table 2. Twelve out of the 14 *p* values (2 genotypes × 7 SNPs) reach a significant level after Bonferroni correction (all *p* values < 0.0125). After controlling cofactors with the Quade test, only 6 *p* values remain significant; however, significance remains observed for at least one genotype type for all SNPs except rs12703526. Summary of *p* values and effect size for between‐groups and within‐groups analyses of 7 selected SNPs after controlling for covariates are shown in Table S3. Overall, the NCs group performs better than the PD group, regardless of their genotype.

#### 3.2.2 RMET Positive Subscore

Table 3 depicts the mean and standard deviation of the positive subscore for each subgroup of the 7 selected SNPs. For subgroup comparisons within NCs and within PD, there are no significant differences before or after covariate adjustment.

**TABLE 3:** Comparison of RMET positive subscore between and within groups of 7 selected SNPs.

	NCs		PD		Stat.^a^	*p* ^a^	Stat.^b^	*p* ^b^
	Mean	SD	Mean	SD				
rs12703526								
TT+TG	3.757	1.891	2.959	1.369	2.499	0.012*	0.466	0.496
GG	3.699	1.571	3.388	1.685	1.755	0.079	0.068	0.795
Stat.^c^	0.252		1.336					
*p* value^c^	0.801		0.182					
Stat.^d^	0.310		0.639					
*p* value^d^	0.578		0.425					
rs11771145								
AA+AG	3.766	1.664	3.243	1.608	2.949	0.003*	1.104	0.294
GG	3.570	1.671	3.250	1.574	0.803	0.422	0.109	0.742
Stat.^c^	−0.943		0.278					
*p* value^c^	0.346		0.781					
Stat.^d^	1.125		0.124					
*p* value^d^	0.289		0.725					
rs7805776								
AA+AG	3.743	1.724	3.232	1.627	2.644	0.008*	0.426	0.515
GG	3.676	1.583	3.269	1.548	1.396	0.163	0.092	0.763
Stat.^c^	−0.015		0.574					
*p* value^c^	0.988		0.566					
Stat.^d^	0.007		0.440					
*p* value^d^	0.933		0.508					
rs9640385								
TT+TC	3.793	1.910	2.952	1.453	2.936	0.003*	2.219	0.138
CC	3.662	1.495	3.464	1.668	1.192	0.233	0.215	0.643
Stat.^c^	−0.338		1.709					
*p* value^c^	0.736		0.087					
Stat.^d^	0.025		4.060					
*p* value^d^	0.875		0.046					
rs9640386								
AA+AG	3.848	1.662	3.151	1.543	3.113	0.002*	2.525	0.113
GG	3.574	1.659	3.338	1.649	0.972	0.331	0.528	0.468
Stat.^c^	−1.780		0.740					
*p* value^c^	0.075		0.459					
Stat.^d^	3.453		0.349					
*p* value^d^	0.064		0.556					
rs2966700								
CC+CT	3.879	1.778	3.030	1.481	4.064	< 0.001*	5.909	0.016
TT	3.476	1.459	3.688	1.740	−0.631	0.528	5.271	0.023
Stat.^c^	−2.306		2.073					
*p* value^c^	0.021		0.038					
Stat.^d^	5.516		3.855					
*p* value^d^	0.019		0.052					
rs2949770								
CC+CA	3.515	1.468	3.697	1.551	−0.551	0.582	1.373	0.243
AA	3.795	1.734	3.114	1.590	3.582	< 0.001*	1.940	0.165
Stat.^c^	1.350		−1.909					
*p* value^c^	0.177		0.056					
Stat.^d^	1.171		3.508					
*p* value^d^	0.280		0.063					

*Note:* The two subgroups are divided by carrying minor allele or not, shown as minor/minor+minor/major subgroup and major/major subgroup in this table (e.g., TT+TG subgroup and GG subgroup in rs12703526).

Abbreviations: NCs, normal controls; PD, Parkinson's disease; RMET, Reading the Mind in the Eyes Test; SD, standard deviation; SNP, single nucleotide polymorphism; Stat., statistical value.

^a^ Comparison between NCs and PD who carry the same genotype using Mann–Whitney *U* test.

^b^ Comparison between NCs and PD who carry the same genotype using Quade test (sex, age, education, and Mini‐Mental State Examination score as covariates).

^c^ Comparison of two subgroups (with and without carrying minor allele) within NCs and within PD using Mann–Whitney *U* test.

^d^ Comparison of two subgroups (with and without carrying minor allele) within NCs and within PD using Quade test (sex, age, education, and Mini‐Mental State Examination score as covariates for NCs group; sex, age, education, Hoehn‐Yahr stage, levodopa equivalent daily dose, and Mini‐Mental State Examination score as covariates for PD group).

* *p* < 0.0125.

For the between‐group comparison, there are significant differences in at least one genotype at all SNPs, except rs12703526, before controlling covariates. After controlling covariates, no difference in RMET‐positive subscores between NCs and PDs is observed.

#### 3.2.3 RMET Negative Subscore

The mean score and standard deviation of the RMET negative subscore for each subgroup of the 7 selected SNPs are shown in Table 4. For between‐group comparisons, 13 out of the 14 *p* values reach the significant level before controlling for covariates, while 5 remain significant after controlling for covariates. The results are similar to the results of the RMET total score, both showing extensive differences in nonspecific genotypes.

**TABLE 4:** Comparison of RMET negative subscore between and within groups of 7 selected SNPs.

	NCs		PD		Stat.^a^	*p* ^a^	Stat.^b^	*p* ^b^
	Mean	SD	Mean	SD				
rs12703526								
TT+TG	6.990	2.391	5.571	2.151	3.413	0.001*	3.462	0.065
GG**	6.900	2.153	5.878	1.933	4.190	< 0.001*	6.691	0.010*
Stat.^c^	−0.207		0.937					
*p* value^c^	0.836		0.349					
Stat.^d^	0.006		0.441					
*p* value^d^	0.940		0.508					
rs11771145								
AA+AG**	6.933	2.232	5.874	1.978	4.418	< 0.001*	8.927	0.003*
GG	6.903	2.197	5.472	2.091	3.186	0.001*	1.618	0.206
Stat.^c^	−0.192		−1.041					
*p* value^c^	0.848		0.298					
Stat.^d^	0.045		1.134					
*p* value^d^	0.832		0.289					
rs7805776								
AA+AG	6.930	2.237	5.895	1.987	3.930	< 0.001*	6.251	0.013
GG	6.919	2.202	5.558	2.043	3.772	< 0.001*	4.039	0.046
Stat.^c^	0.027		−0.952					
*p* value^c^	0.979		0.341					
Stat.^d^	0.001		1.051					
*p* value^d^	0.977		0.307					
rs9640385								
TT+TC**	7.264	2.281	5.730	2.209	4.276	< 0.001*	9.131	0.003*
CC	6.708	2.171	5.810	1.853	3.447	0.001*	2.128	0.146
Stat.^c^	−1.890		0.499					
*p* value^c^	0.059		0.618					
Stat.^d^	2.050		0.214					
*p* value^d^	0.153		0.644					
rs9640386								
AA+AG	6.876	2.154	5.795	1.936	3.577	< 0.001*	4.683	0.031
GG	6.945	2.262	5.757	2.086	4.014	< 0.001*	4.840	0.029
Stat.^c^	0.325		−0.325					
*p* value^c^	0.745		0.745					
Stat.^d^	0.380		0.002					
*p* value^d^	0.538		0.969					
rs2966700								
CC+CT**	7.009	2.168	5.677	1.994	5.133	< 0.001*	13.140	< 0.001*
TT	6.803	2.295	5.979	2.037	2.158	0.031	0.146	0.703
Stat.^c^	−0.764		0.977					
*p* value^c^	0.445		0.328					
Stat.^d^	0.379		2.522					
*p* value^d^	0.538		0.114					
rs2949770								
CC+CA	6.757	1.943	5.636	2.044	2.529	0.011*	1.157	0.284
AA**	6.992	2.321	5.816	2.003	4.798	< 0.001*	9.460	0.002*
Stat.^c^	1.068		0.289					
*p* value^c^	0.286		0.773					
Stat.^d^	1.415		0.003					
*p* value^d^	0.235		0.953					

*Note:* The two subgroups are divided by carrying minor allele or not, shown as minor/minor+minor/major subgroup and major/major subgroup in this table (e.g., eg. TT+TG subgroup and GG subgroup in rs12703526). The number of participants in different genotype subgroups are listed in Table S2.

Abbreviations: NCs, normal controls; PD, Parkinson's disease; RMET, Reading the Mind in the Eyes Test; SD, standard deviation; SNP, single nucleotide polymorphism; Stat., statistical value.

^a^ Comparison between NCs and PD who carry the same genotype using Mann–Whitney *U* test.

^b^ Comparison between NCs and PD who carry the same genotype using Quade test (sex, age, education, and Mini‐Mental State Examination score as covariates).

^c^ Comparison of two subgroups (with and without carrying minor allele) within NCs and within PD using Mann–Whitney *U* test.

^d^ Comparison of two subgroups (with and without carrying minor allele) within NCs and within PD using Quade test (sex, age, education, and Mini‐Mental State Examination score as covariates for NCs group; sex, age, education, Hoehn‐Yahr stage, levodopa equivalent daily dose, and Mini‐Mental State Examination score as covariates for PD group).

* *p* < 0.0125.

** Genotypes that showed a significant RMET difference between NCs and PD after controlling sex, age, education level, and Mini‐Mental State Examination score.

For subgroup comparisons within NCs and within PD, no SNPs reach significance either before or after controlling for covariates.

#### 3.2.4 RMET Neutral Subscore

The mean score and standard deviation of the RMET negative subscore for each subgroup of the 7 selected SNPs are shown in Table S4. For between‐group comparisons, we can see that 11 of the 14 *p* values reach the significance level before controlling for covariates. After controlling for the covariates, only rs2966700 CC+CT remains significantly different.

For subgroup comparisons within NCs and within PD, no SNPs reach significance before or after controlling for covariates.

#### 3.2.5 Statistical Power

In between‐group comparisons, the minor‐allele group at rs12703526 and rs2949770, and the major‐allele group at rs11771145, had lower power. All subgroup comparisons within PD showed lower statistical power. Detailed values are listed in Table S5. Although most of the above comparisons are insignificant after controlling for covariates, the smaller sample size may obscure significance, and the results should be interpreted with caution.

#### 3.2.6 Moderation Analysis and Main Genetic Effect

**FIGURE 1:**
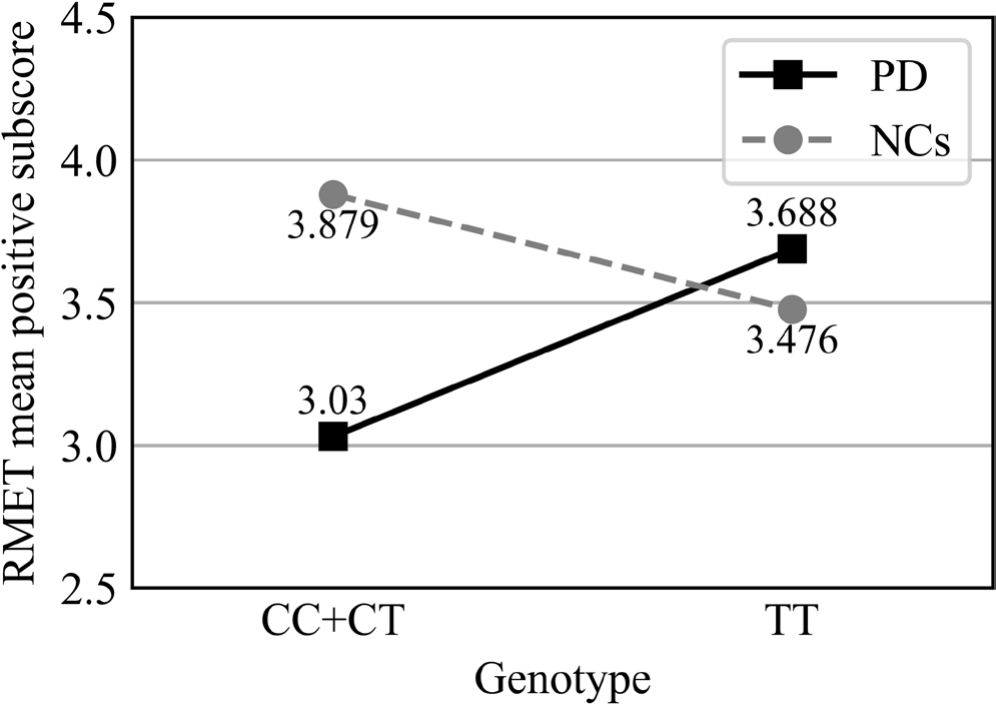
The moderation effect of disease and rs2966700 on RMET mean positive subscore. It depicts the mean positive subscore of participants carrying different genotype on rs2966700. The difference between PD and NCs is more pronounced in the CC+CT group (the *p* value before and after controlling covariates are *p* < 0.001 and *p* = 0.016, respectively) compared with the gap in the TT group (the *p* value before and after controlling covariates are *p* = 0.528 and *p* = 0.023, respectively), indicating a significant interplay between gene and disease in terms of RMET score. This moderation effect is significantly seen (*p* = 0.001). *p value < 0.0125.

We conduct a moderation analysis of the main genetic effect of the 7 SNPs, using the RMET total score, positive subscore, negative subscore, and neutral subscore as dependent variables. The detailed numbers are shown in Table 5 and Table S6. Rs2966700 has a significant main genetic effect on the RMET positive subscore in the whole cohort (*p* = 0.006, partial *R* ^2^ = 0.015), and those who carry the TT genotype exhibit better social cognition than those who carry the C allele. Additionally, only rs2966700 shows a moderation effect of PD on RMET positive subscore (*p* = 0.001, *R* ^2^ change = 0.020), indicating an interaction between rs2966700 and the groups (NCs and PD). The moderation effect and the difference between PD and NCs are shown in Figure 1.

**TABLE 5:** Main effect of gene and moderation effect derived from regression analysis^a^.

	Main effect of gene		Moderation		Effect in NCs		Effect in PD	
	Stat.	*p*	Stat.	*p*	Stat.	*p*	Stat.	*p*
rs12703526								
Total	0.753	0.452	0.047	0.828				
Positive	1.303	0.193	1.324	0.250				
Negative	0.679	0.498	0.460	0.498				
Neutral	0.545	0.586	0.635	0.426				
rs11771145								
Total	−1.799	0.073	2.054	0.152				
Positive	0.355	0.723	0.692	0.406				
Negative	−0.693	0.489	0.270	0.604				
Neutral	−1.942	0.053	1.996	0.158				
rs7805776								
Total	−1.434	0.152	2.133	0.145				
Positive	0.249	0.803	0.200	0.655				
Negative	−0.673	0.501	0.256	0.613				
Neutral	−1.502	0.134	1.593	0.207				
rs9640385								
Total	0.836	0.404	0.970	0.325				
Positive	2.066	0.039	3.479	0.063				
Negative	0.855	0.393	2.524	0.113				
Neutral	−0.003	0.998	0.218	0.641				
rs9640386								
Total	0.521	0.603	1.243	0.265				
Positive	0.933	0.351	2.221	0.137				
Negative	−0.237	0.813	0.204	0.651				
Neutral	0.102	0.919	0.108	0.743				
rs2966700								
Total	1.598	0.111	4.007	0.046				
Positive	2.745	0.006*	10.901	0.001*	−2.172	0.030	2.538	0.012*
Negative	1.156	0.248	1.645	0.200				
Neutral	0.388	0.698	1.021	0.312				
rs2949770								
Total	−0.123	0.902	0.023	0.880				
Positive	−1.996	0.046	5.417	0.020				
Negative	0.594	0.553	0.008	0.930				
Neutral	0.601	0.548	0.587	0.444				

Abbreviations: NCs, normal controls; PD, Parkinsons's disease; Stat., statistical value.

^a^ Sex, age, education, and Mini‐Mental State Examination score as covariates.

* *p* < 0.0125.

## 4 Discussion

This study aims to elucidate the complex relationship between social cognition and the *EPHA1‐AS1* gene in patients with PD and NCs using RMET scores. Our analysis revealed differences between PD and NCs across multiple SNPs, particularly in RMET total and negative subscores, and identified both a main genetic effect and a disease‐moderated effect at rs2966700 on the RMET positive subscore. In essence, the *EPHA1‐AS1* gene appears to play an interactive role in Parkinson's disease, influencing social cognition. Although the observed effect sizes were small, as shown in Table S3 and Table 5, this is consistent with prior genetic studies of cognitive phenotypes, in which individual common variants are expected to exert modest effects. Cognitive performance is a distal behavioral outcome influenced by polygenic and environmental factors, and effect sizes may be attenuated by measurement variability, covariate adjustment, and clinical heterogeneity. Importantly, the presence of a significant genotype‐by‐disease interaction supports disease‐specific genetic modulation rather than a spurious association.

The pervasive differences between PD and NCs in numerous SNPs suggest that the substantial impact of PD itself may overshadow the genetic contributions. This reaffirms earlier findings [28], and consolidates the notion that PD patients exhibit poorer social cognition than NCs. Impairment in specific brain regions is a plausible explanation for the observed deficits in social cognition among patients with PD. Notably, prior research has shown that the limbic loop is important for social cognition [49]. The brain impairment and decreased dopamine level in the limbic loop are hypothesized to have an impact on the ToM in PD patients [5, 50, 51].

The difference between NCs and PD is more pronounced in the RMET total score and the negative subscore. While existing studies diverge on whether PD patients specifically manifest deficits in certain emotions, most studies illuminate deficits across negative emotions, which is corresponsive to our results. The underlying reason for this might be the high prevalence of hypomimia in PD, which is related to the inability to produce and recognize negative emotions [14]. Besides, reduced gray matter volume and functional connectivity in brain areas for processing and interpreting negative affective cues [52] in PD might also explain the deficit.

Rs2966700 is located at chromosome 7, position 143,510,144 (GRCh38). The genetic influence of rs2966700 on social cognition is significant (*p* = 0.006, partial *R* ^2^ = 0.015), with individuals carrying the TT genotype demonstrating enhanced social cognition compared to those with the C allele. Furthermore, the interaction between rs2966700 and disease is noteworthy, particularly for the RMET positive subscore (*p* = 0.001, *R* ^2^ = 0.020).

Previous studies have shown that the EPHA1 protein might be involved in inflammation and the development of AD and PD. EPHA1 has been implicated in neuroinflammation by activating the CXCL12/CXCR4 pathway in both PD mouse and cell models [37]. This pathway contributes to microglial accumulation and α‐synuclein–induced inflammation [40]. Notably, CXCR4‐knockout in MPTP‐lesioned mice attenuated dopaminergic neurodegeneration and glial activation [39], highlighting the EPHA1–CXCL12/CXCR4 axis as a potential mechanism in PD pathogenesis.

We therefore interpret our findings as suggesting that the rs2966700 C allele is associated with reduced *EPHA1‐AS1* activity, resulting in less inhibition of EPHA1 translation and consequently greater neuroinflammation and poorer social cognition. The observed genotype‐by‐disease interaction further indicates that the C allele may amplify PD‐related neuroinflammatory effects, as between‐group differences were evident among C allele carriers but not in the TT group. Although no direct molecular data are available in our current study, multiple lines of evidence support the plausibility of an *EPHA1‐AS1*‐mediated inflammatory mechanism. Rs11765305, a variant of *EPHA1*, is a functional eQTL that upregulates *EPHA1‐AS1* and may enhance activity of the JAK2/STAT3 signaling axis [53], which is known to regulate immune responses. Transcriptomic studies in psoriatic skin samples reveal significant upregulation of *EPHA1‐AS1*, suggesting its involvement in inflammatory regulation in peripheral tissues [43].

Besides its inflammatory function, EPHA1 was found to play roles in cell–cell communication, synaptic plasticity, and the integrity of the BBB, processes that have been implicated in the pathogenesis of AD and PD [38, 41]. Alpha‐synuclein aggregation and genetic disruption of synaptic proteins might drive BBB dysfunction and early synaptic dysfunction, which precedes neuronal cell death and contributes to both motor and cognitive symptoms by impairing dopaminergic and excitatory synapses [54, 55, 56]. Variation of rs2966700 might alter the function of *EPHA1* and further lead to the pathological and clinical results of PD, including social cognition impairment. However, our interpretation of the results remains exploratory and speculative, and further direct molecular experiments are needed to verify them.

Although *EPHA1‐AS1* genotypes did not show a main effect on RMET performance within either NCs or PD groups, a significant disease‐by‐genotype (rs2966700) interaction was observed for positive social cognition, suggesting that rs2966700 might affect social cognition through modulation of individual susceptibility to PD‐related impairment instead of direct determination. The absence of genotype differences within PD may indicate that the disease‐related effect on social cognition is sufficiently strong to override the genotype‐specific effects at the within‐group level. Notably, this attenuation does not preclude the detection of genotype‐dependent effects in more sensitive social cognitive domains. We suggest that the reason why rs2966700 genetic effect and disease‐moderated effect can only be observed in the positive subscore is possibly due to higher sensitivity of the positive subscore. This raises intriguing questions regarding the nuanced nature of positive emotion recognition in the context of social cognition. Positive emotion recognition relies on finely integrated social–emotional and reward‐related neural networks, which may render it particularly vulnerable to subtle disruptions in pathological processes in which the EPHA1 is involved. Previous MRI study showed that the right medial orbitofrontal region is associated with happiness recognition [57], which is an area involved in the reward system [58], a pathway important for the formation and recognition of positive emotions. Dysfunction in these areas might explain positive emotion vulnerability. Although there is still no consensus on the brain areas responsible for specific emotion recognition, a review has shown that positive emotions seem to engage more cortical areas than negative emotions do [59], suggesting a greater dependence on distributed cortical networks and, consequently, increased susceptibility to inflammation‐related or synaptic functional alterations. The mechanisms are hypothetical, and future studies integrating genetic, molecular, neuroimaging, and behavioral data are warranted to validate them.

Although seven SNPs were examined, only rs2966700 showed a significant association with social cognition in PD. The absence of significant findings for the remaining SNPs may result from limited statistical power, reducing sensitivity to detect modest effects after multiple‐testing correction, as well as the subtle, context‐dependent nature of genetic influences on social cognition and locus‐specific biological effects whereby only certain variants are functionally relevant. Accordingly, these null findings should be interpreted cautiously and may indicate heterogeneous genetic contributions to social cognition in PD.

Limitations of this study include reliance solely on RMET for social cognition evaluation. Previous study has shown that RMET is a test specifically for emotion recognition rather than overall ToM [60]. To yield a more comprehensive understanding of cognitive ToM, future studies that employ multiple assessment methods, such as false‐belief tasks, faux pas tests, etc., simultaneously are needed. Second, lower statistical power was observed in some of our analyses, which may be due to fewer participants, alpha‐level correction for multiple analyses, and more covariates controlled for in our study. Additionally, the exclusive inclusion of PD and NCs may limit the generalizability of our findings. Future investigations could enrich our understanding by expanding the participant groups to include individuals with conditions characterized by inflammation, such as hepatitis or cancer patients, as we suggest that the effect of the *EPHA1‐AS1* gene on PD is mediated through inflammation. Lastly, though no participants reported being diagnosed with depression, possibly existing depressive symptoms were not controlled in our analysis due to the lack of complete depressive questionnaire data, which could lead to bias in our results.

In conclusion, the study reaffirms previous findings, highlighting a deficit of negative emotions across the entire PD cohort and revealing the interactive influence of rs2966700 and disease on the RMET positive subscore, suggesting that the C allele may exacerbate neuroinflammation in PD patients. Pioneering the exploration of the *EPHA1‐AS1* gene in PD patients, our study provides valuable insights into the non‐motor facets of PD. Further molecular studies are needed to uncover the mechanisms behind this in the future. From a translational perspective, rs2966700 may serve as a potential biomarker to identify individuals at increased risk of social cognitive impairment in Parkinson's disease, informing future studies on early or targeted intervention strategies, including those targeting neuroinflammatory mechanisms.

## Author Contributions

Yu‐Chen Lin: conceptualization, methodology, formal analysis, writing‐original draft, visualization, funding acquisition. Chun‐Hsiang Tan: conceptualization, methodology, investigation, resources, data curation, writing‐review, and editing. Wei‐Pin Hong: investigation, resources, writing‐review, and editing. Rwei‐Ling Yu: conceptualization, methodology, data curation, writing‐review and editing, supervision, project administration, funding acquisition.

## Funding

This study was funded by the Ministry of Science and Technology (MOST), Taipei, Taiwan (MOST 110‐2628‐B‐006‐020 and MOST 111‐2628‐B‐006‐020), the National Science and Technology Council, Taipei, Taiwan (NSTC 112‐2628‐B‐006‐002 and NSTC 113‐2410‐H‐006‐093‐MY2), National Cheng Kung University Hospital, Tainan, Taiwan (NCKUH‐11210045), and the Summer Research Project Grant no. NCKUMCS2021037 from the College of Medicine at National Cheng Kung University.

## Ethics Statement

This study was conducted in accordance with the ethical principles of the Declaration of Helsinki. All participants provided consent prior to the examinations. All study procedures were approved by the Ethics Research Committee of National Cheng Kung University Hospital (approval number: A‐ER‐109‐491).

## Conflicts of Interest

The authors declare no conflicts of interest.

## Supporting information


**Table S1:** Included SNPs of *EPHA1‐AS1* gene for the statistical analyses of this study.

**Table S2:** Number of participants.

**Table S3:** Summary of *p* values and effect size for between‐groups and within‐groups analyses of 7 selected SNPs after controlling covariates.

**Table S4:** Comparison of RMET neutral subscore between and within groups of 7 selected SNPs.

**Table S5:** Statistical power of comparisons between and within groups after controlling covariates.

**Table S6:** Results of the moderation effect of rs2966700 on RMET positive subscore derived from the regression analysis.

## Data Availability

The datasets used and/or analyzed during the current study are available from the corresponding author on reasonable request.
