# Supplementary material for: Deciphering the Impact of EPHA1‐AS1 Gene Polymorphism on Social Cognition Deficits in Parkinson's Disease
Source: CNS Neurosci Ther. 2026 Mar 27;32(4):e70801. doi: 10.1002/cns.70801 (PMC13140347; doi:10.1002/cns.70801)
Supplement: Supplementary file 1 — Table S1: Included SNPs of EPHA1‐AS1 gene for the statistical analyses of this study. [file CNS-32-e70801-s002.docx]

| **Supplementary Table 1.** Included SNPs of *EPHA1-AS1* gene for the statistical analyses of this study. | | | | | | |
| --- | --- | --- | --- | --- | --- | --- |
| SNP | Position | Minor/major allele | MAF | HWE *p* value | Block ^†^ | Included in the analysis |
| rs12703526 | 143410495 | T/G | 0.160 | 0.639 | 1 | Y |
| rs11763230 | 143411748 | T/C | 0.129 | >0.999 | 1 |  |
| rs11767557 | 143412046 | C/T | 0.129 | >0.999 | 1 |  |
| rs11771145 | 143413669 | A/G | 0.494 | 0.909 | 2 | Y |
| rs78432897 | 143414822 | G/C | 0.275 | 0.867 | 2 |  |
| rs7805776 | 143427203 | A/G | 0.376 | 0.765 | 3 | Y |
| rs62474772 | 143432213 | T/C | 0.115 | 0.148 | 4 |  |
| rs9640385 | 143433224 | T/C | 0.226 | >0.999 | 4 | Y |
| rs9640386 | 143435090 | A/G | 0.288 | >0.999 | 5 | Y |
| rs2140786 | 143457092 | A/G | 0.230 | 0.560 | 6 |  |
| rs12666496 | 143471353 | T/A | 0.113 | >0.999 | 6 |  |
| rs12669468 | 143508014 | A/G | 0.102 | >0.999 | 6 |  |
| rs2966700 | 143510144 | C/T | 0.389 | 0.391 | 6 | Y |
| rs2966699 | 143511121 | A/C | 0.107 | >0.999 | 6 |  |
| rs10260451 | 143511132 | A/G | 0.229 | 0.460 | 6 |  |
| rs17382409 | 143511875 | G/A | 0.282 | 0.857 | 6 |  |
| Affx-29875906 | 143515714 | C/T | 0.388 | 0.429 | 6 |  |
| rs2949770 | 143520097 | C/A | 0.143 | >0.999 | 7 | Y |
| Abbreviations: SNP, single-nucleotide polymorphism; HWE, Hardy-Weinberg equilibrium; MAF, Minor allele frequency. ^†^Block is derived from the linkage disequilibrium results, which are generated through Haploview version 4.2 using the definition “solid spine of LD” for the analysis. Y: SNPs that have the highest minor allele frequency in each block are included in further analysis. | | | | | | |
