# Supplementary material for: Deciphering the Impact of EPHA1‐AS1 Gene Polymorphism on Social Cognition Deficits in Parkinson's Disease
Source: CNS Neurosci Ther. 2026 Mar 27;32(4):e70801. doi: 10.1002/cns.70801 (PMC13140347; doi:10.1002/cns.70801)
Supplement: Supplementary file 2 — Table S2: Number of participants. [file CNS-32-e70801-s003.docx]

| **Supplementary Table 2.** Number of participants | | | |
| --- | --- | --- | --- |
|  | NCs | PD | total |
| ***rs12703526*** |  |  |  |
| *TT+TG* | 103 | 49 | 152 |
| *GG* | 259 | 98 | 357 |
| total | 362 | 147 | 509 |
| ***rs11771145*** |  |  |  |
| *AA+AG* | 269 | 111 | 380 |
| *GG* | 93 | 36 | 129 |
| total | 362 | 147 | 509 |
| ***rs7805776*** |  |  |  |
| *AA+AG* | 214 | 95 | 309 |
| *GG* | 148 | 52 | 200 |
| total | 362 | 147 | 509 |
| ***rs9640385*** |  |  |  |
| *TT+TC* | 140 | 63 | 203 |
| *CC* | 219 | 84 | 303 |
| total | 359 | 147 | 506 |
| ***rs9640386*** |  |  |  |
| *AA+AG* | 178 | 73 | 251 |
| *GG* | 183 | 74 | 257 |
| total | 361 | 147 | 508 |
| ***rs2966700*** |  |  |  |
| *CC+CT* | 215 | 99 | 314 |
| *TT* | 147 | 48 | 195 |
| total | 362 | 147 | 509 |
| ***rs2949770*** |  |  |  |
| *CC+CA* | 103 | 33 | 136 |
| *AA* | 259 | 114 | 373 |
| total | 362 | 147 | 509 |
| Abbreviations: normal controls, NCs; Parkinson’s disease, PD | | | |
