# Supplementary material for: Deciphering the Impact of EPHA1‐AS1 Gene Polymorphism on Social Cognition Deficits in Parkinson's Disease
Source: CNS Neurosci Ther. 2026 Mar 27;32(4):e70801. doi: 10.1002/cns.70801 (PMC13140347; doi:10.1002/cns.70801)
Supplement: Supplementary file 3 — Table S3: Summary of p values and effect size for between‐groups and within‐groups analyses of 7 selected SNPs after controlling covariates. [file CNS-32-e70801-s005.docx]

**Supplementary Table 3.** Summary of p values and effect size for between-groups and within-groups analyses of 7 selected SNPs after controlling covariates

| SNP | RMET domain | Comparison (group) | *p* value | effect size (partial η²) for significant p values |
| --- | --- | --- | --- | --- |
| rs12703526 | Total | NCs vs PD (TT+TG) | 0.110 |  |
|  |  | NCs vs PD (GG) | 0.015 |  |
|  |  | TT+TG vs GG (NCs) | 0.723 |  |
|  |  | TT+TG vs GG (PD) | 0.397 |  |
|  | Positive | NCs vs PD (TT+TG) | 0.496 |  |
|  |  | NCs vs PD (GG) | 0.795 |  |
|  |  | TT+TG vs GG (NCs) | 0.578 |  |
|  |  | TT+TG vs GG (PD) | 0.425 |  |
|  | Negative | NCs vs PD (TT+TG) | 0.065 |  |
|  |  | NCs vs PD (GG) | 0.010* | 0.019 |
|  |  | TT+TG vs GG (NCs) | 0.940 |  |
|  |  | TT+TG vs GG (PD) | 0.508 |  |
|  | Neutral | NCs vs PD (TT+TG) | 0.047 |  |
|  |  | NCs vs PD (GG) | 0.116 |  |
|  |  | TT+TG vs GG (NCs) | 0.411 |  |
|  |  | TT+TG vs GG (PD) | 0.627 |  |
| rs11771145 | Total | NCs vs PD (AA+AG) | 0.009* | 0.018 |
|  |  | NCs vs PD (GG) | 0.110 |  |
|  |  | AA+AG vs GG (NCs) | 0.906 |  |
|  |  | AA+AG vs GG (PD) | 0.140 |  |
|  | Positive | NCs vs PD (AA+AG) | 0.294 |  |
|  |  | NCs vs PD (GG) | 0.742 |  |
|  |  | AA+AG vs GG (NCs) | 0.289 |  |
|  |  | AA+AG vs GG (PD) | 0.725 |  |
|  | Negative | NCs vs PD (AA+AG) | 0.003* | 0.023 |
|  |  | NCs vs PD (GG) | 0.206 |  |
|  |  | AA+AG vs GG (NCs) | 0.832 |  |
|  |  | AA+AG vs GG (PD) | 0.289 |  |
|  | Neutral | NCs vs PD (AA+AG) | 0.041 |  |
|  |  | NCs vs PD (GG) | 0.166 |  |
|  |  | AA+AG vs GG (NCs) | 0.749 |  |
|  |  | AA+AG vs GG (PD) | 0.043 |  |
| rs7805776 | Total | NCs vs PD (AA+AG) | 0.051 |  |
|  |  | NCs vs PD (GG) | 0.012* | 0.031 |
|  |  | AA+AG vs GG (NCs) | 0.476 |  |
|  |  | AA+AG vs GG (PD) | 0.232 |  |
|  | Positive | NCs vs PD (AA+AG) | 0.515 |  |
|  |  | NCs vs PD (GG) | 0.763 |  |
|  |  | AA+AG vs GG (NCs) | 0.933 |  |
|  |  | AA+AG vs GG (PD) | 0.508 |  |
|  | Negative | NCs vs PD (AA+AG) | 0.013 |  |
|  |  | NCs vs PD (GG) | 0.046 |  |
|  |  | AA+AG vs GG (NCs) | 0.832 |  |
|  |  | AA+AG vs GG (PD) | 0.289 |  |
|  | Neutral | NCs vs PD (AA+AG) | 0.098 |  |
|  |  | NCs vs PD (GG) | 0.055 |  |
|  |  | AA+AG vs GG (NCs) | 0.728 |  |
|  |  | AA+AG vs GG (PD) | 0.078 |  |
| rs9640385 | Total | NCs vs PD (TT+TC) | 0.010* | 0.032 |
|  |  | NCs vs PD (CC) | 0.077 |  |
|  |  | TT+TC vs CC (NCs) | 0.431 |  |
|  |  | TT+TC vs CC (PD) | 0.384 |  |
|  | Positive | NCs vs PD (TT+TC) | 0.138 |  |
|  |  | NCs vs PD (CC) | 0.643 |  |
|  |  | TT+TC vs CC (NCs) | 0.875 |  |
|  |  | TT+TC vs CC (PD) | 0.046 |  |
|  | Negative | NCs vs PD (TT+TC) | 0.003* | 0.043 |
|  |  | NCs vs PD (CC) | 0.029 |  |
|  |  | TT+TC vs CC (NCs) | 0.538 |  |
|  |  | TT+TC vs CC (PD) | 0.969 |  |
|  | Neutral | NCs vs PD (TT+TC) | 0.103 |  |
|  |  | NCs vs PD (CC) | 0.062 |  |
|  |  | TT+TC vs CC (NCs) | 0.438 |  |
|  |  | TT+TC vs CC (PD) | 0.817 |  |
| rs9640386 | Total | NCs vs PD (AA+AG) | 0.002* | 0.037 |
|  |  | NCs vs PD (GG) | 0.300 |  |
|  |  | AA+AG vs GG (NCs) | 0.171 |  |
|  |  | AA+AG vs GG (PD) | 0.690 |  |
|  | Positive | NCs vs PD (AA+AG) | 0.113 |  |
|  |  | NCs vs PD (GG) | 0.468 |  |
|  |  | AA+AG vs GG (NCs) | 0.064 |  |
|  |  | AA+AG vs GG (PD) | 0.556 |  |
|  | Negative | NCs vs PD (AA+AG) | 0.031 |  |
|  |  | NCs vs PD (GG) | 0.029 |  |
|  |  | AA+AG vs GG (NCs) | 0.538 |  |
|  |  | AA+AG vs GG (PD) | 0.969 |  |
|  | Neutral | NCs vs PD (AA+AG) | 0.045 |  |
|  |  | NCs vs PD (GG) | 0.203 |  |
|  |  | AA+AG vs GG (NCs) | 0.527 |  |
|  |  | AA+AG vs GG (PD) | 0.983 |  |
| rs2966700 | Total | NCs vs PD (CC+CT) | <0.001* | 0.046 |
|  |  | NCs vs PD (TT) | 0.854 |  |
|  |  | CC+CT vs TT (NCs) | 0.138 |  |
|  |  | CC+CT vs TT (PD) | 0.066 |  |
|  | Positive | NCs vs PD (CC+CT) | 0.016 |  |
|  |  | NCs vs PD (TT) | 0.023 |  |
|  |  | CC+CT vs TT (NCs) | 0.019 |  |
|  |  | CC+CT vs TT (PD) | 0.052 |  |
|  | Negative | NCs vs PD (CC+CT) | <0.001* | 0.040 |
|  |  | NCs vs PD (TT) | 0.703 |  |
|  |  | CC+CT vs TT (NCs) | 0.538 |  |
|  |  | CC+CT vs TT (PD) | 0.114 |  |
|  | Neutral | NCs vs PD (CC+CT) | 0.009* | 0.022 |
|  |  | NCs vs PD (TT) | 0.503 |  |
|  |  | CC+CT vs TT (NCs) | 0.241 |  |
|  |  | CC+CT vs TT (PD) | 0.795 |  |
| rs2949770 | Total | NCs vs PD (CC+CA) | 0.293 |  |
|  |  | NCs vs PD (AA) | 0.003* | 0.023 |
|  |  | CC+CA vs AA (NCs) | 0.926 |  |
|  |  | CC+CA vs AA (PD) | 0.701 |  |
|  | Positive | NCs vs PD (CC+CA) | 0.243 |  |
|  |  | NCs vs PD (AA) | 0.165 |  |
|  |  | CC+CA vs AA (NCs) | 0.280 |  |
|  |  | CC+CA vs AA (PD) | 0.063 |  |
|  | Negative | NCs vs PD (CC+CA) | 0.284 |  |
|  |  | NCs vs PD (AA) | 0.002* | 0.025 |
|  |  | CC+CA vs AA (NCs) | 0.235 |  |
|  |  | CC+CA vs AA (PD) | 0.953 |  |
|  | Neutral | NCs vs PD (CC+CA) | 0.137 |  |
|  |  | NCs vs PD (AA) | 0.062 |  |
|  |  | CC+CA vs AA (NCs) | 0.263 |  |
|  |  | CC+CA vs AA (PD) | 0.504 |  |
| Abbreviations: SNP, single nucleotide polymorphism; NCs, normal controls; PD, Parkinson’s disease; vs, versus; the two subgroups are divided by carrying minor allele or not, shown as minor/minor+minor/major subgroup and major/major subgroup in this table (eg. TT+TG subgroup and GG subgroup in rs12703526)  ^†^Comparison between NCs and PD who carry the same genotype using Quade test (sex, age, education, Mini-Mental State Examination score as covariates) and comparison of two subgroups (with and without carrying minor allele) within NCs and within PD using Quade test (sex, age, education, Mini-Mental State Examination score as covariates for NCs group; sex, age, education, Hoehn-Yahr stage, levodopa equilivant daily dose, Mini-Mental State Examination score as covariates for PD group)  *p<0.0125 | | | | |
