# Supplementary material for: Deciphering the Impact of EPHA1‐AS1 Gene Polymorphism on Social Cognition Deficits in Parkinson's Disease
Source: CNS Neurosci Ther. 2026 Mar 27;32(4):e70801. doi: 10.1002/cns.70801 (PMC13140347; doi:10.1002/cns.70801)
Supplement: Supplementary file 4 — Table S4: Comparison of RMET neutral subscore between and within groups of 7 selected SNPs. [file CNS-32-e70801-s004.docx]

| **Supplementary Table 4.** Comparison of RMET neutral subscore between and within groups of 7 selected SNPs | | | | | | | | |
| --- | --- | --- | --- | --- | --- | --- | --- | --- |
|  | NCs | | PD | | Stat.^†^ | *p* value^†^ | Stat.^‡^ | *p* value^‡^ |
|  | mean | SD | mean | SD |  |  |  |  |
| ***rs12703526*** |  |  |  |  |  |  |  |  |
| TT+TG | 10.320 | 2.687 | 8.959 | 2.423 | 3.099 | 0.002* | 4.027 | 0.047 |
| GG | 10.108 | 2.309 | 9.194 | 2.486 | 2.844 | 0.004* | 2.488 | 0.116 |
| Stat.^§^ | -0.895 | | 0.640 | |  |  |  |  |
| *p* value^§^ | 0.371 | | 0.522 | |  |  |  |  |
| Stat.^¶^ | 0.678 | | 0.237 | |  |  |  |  |
| *p* value^¶^ | 0.411 | | 0.627 | |  |  |  |  |
| ***rs11771145*** |  |  |  |  |  |  |  |  |
| AA+AG | 10.208 | 2.407 | 9.378 | 2.390 | 2.939 | 0.003* | 4.192 | 0.041 |
| GG | 10.054 | 2.469 | 8.306 | 2.528 | 3.265 | 0.001* | 1.942 | 0.166 |
| Stat.^§^ | -0.378 | | -2.176 | |  |  |  |  |
| *p* value^§^ | 0.706 | | 0.030 | |  |  |  |  |
| Stat.^¶^ | 0.103 | | 4.164 | |  |  |  |  |
| *p* value^¶^ | 0.749 | | 0.043 | |  |  |  |  |
| ***rs7805776*** |  |  |  |  |  |  |  |  |
| AA+AG | 10.164 | 2.441 | 9.389 | 2.464 | 2.332 | 0.020 | 2.753 | 0.098 |
| GG | 10.176 | 2.398 | 8.615 | 2.394 | 3.794 | <0.001* | 3.714 | 0.055 |
| Stat.^§^ | 0.277 | | -1.961 | |  |  |  |  |
| *p* value^§^ | 0.782 | | 0.050 | |  |  |  |  |
| Stat.^¶^ | 0.121 | | 3.148 | |  |  |  |  |
| *p* value^¶^ | 0.728 | | 0.078 | |  |  |  |  |
| ***rs9640385*** |  |  |  |  |  |  |  |  |
| TT+TC | 10.421 | 2.496 | 9.286 | 2.593 | 2.751 | 0.006* | 2.682 | 0.103 |
| CC | 10.023 | 2.353 | 8.988 | 2.362 | 3.239 | 0.001* | 3.514 | 0.062 |
| Stat.^§^ | -1.297 | | -0.634 | |  |  |  |  |
| *p* value^§^ | 0.195 | | 0.526 | |  |  |  |  |
| Stat.^¶^ | 0.602 | | 0.054 | |  |  |  |  |
| *p* value^¶^ | 0.438 | | 0.817 | |  |  |  |  |
| ***rs9640386*** |  |  |  |  |  |  |  |  |
| AA+AG | 10.270 | 2.269 | 9.068 | 2.335 | 3.436 | 0.001* | 4.056 | 0.045 |
| GG | 10.055 | 2.559 | 9.162 | 2.591 | 2.398 | 0.016 | 1.627 | 0.203 |
| Stat.^§^ | -0.876 | | 0.156 | |  |  |  |  |
| *p* value^§^ | 0.381 | | 0.876 | |  |  |  |  |
| Stat.^¶^ | 0.400 | | <0.001 | |  |  |  |  |
| *p* value^¶^ | 0.527 | | 0.983 | |  |  |  |  |
| ***rs2966700*** |  |  |  |  |  |  |  |  |
| CC+CT** | 10.358 | 2.373 | 9.131 | 2.402 | 3.876 | <0.001* | 6.945 | 0.009* |
| TT | 9.891 | 2.469 | 9.083 | 2.600 | 1.859 | 0.063 | 0.450 | 0.503 |
| Stat.^§^ | -1.291 | | 0.171 | |  |  |  |  |
| *p* value^§^ | 0.197 | | 0.864 | |  |  |  |  |
| Stat.^¶^ | 1.381 | | 0.068 | |  |  |  |  |
| *p* value^¶^ | 0.241 | | 0.795 | |  |  |  |  |
| ***rs2949770*** |  |  |  |  |  |  |  |  |
| CC+CA | 10.311 | 2.536 | 9.091 | 2.350 | 2.565 | 0.010* | 2.243 | 0.137 |
| AA | 10.112 | 2.375 | 9.123 | 2.500 | 3.193 | 0.001* | 3.501 | 0.062 |
| Stat.^§^ | -1.118 | | 0.396 | |  |  |  |  |
| *p* value^§^ | 0.264 | | 0.692 | |  |  |  |  |
| Stat.^¶^ | 1.259 | | 0.448 | |  |  |  |  |
| *p* value^¶^ | 0.263 | | 0.504 | |  |  |  |  |
| Abbreviations: RMET, Reading the Mind in the Eyes Test; SNP, single nucleotide polymorphism; NCs, normal controls; PD, Parkinson’s disease; Stat., statistical value; SD, standard deviation; the two subgroups are divided by carrying minor allele or not, shown as minor/minor+minor/major subgroup and major/major subgroup in this table (eg. TT+TG subgroup and GG subgroup in rs12703526)  ^†^Comparison between NCs and PD who carry the same genotype using Mann-Whitney U test, ^‡^Comparison between NCs and PD who carry the same genotype using Quade test (sex, age, education, Mini-Mental State Examination score as covariates), ^§^Comparison of two subgroups (with and without carrying minor allele) within NCs and within PD using Mann-Whitney U test, ^¶^Comparison of two subgroups (with and without carrying minor allele) within NCs and within PD using Quade test (sex, age, education, Mini-Mental State Examination score as covariates for NCs group; sex, age, education, Hoehn-Yahr stage, levodopa equilivant daily dose, Mini-Mental State Examination score as covariates for PD group), *p<0.0125,**genotypes that showed a significant RMET difference between NCs and PD after controlling sex, age, education level, and Mini-Mental State Examination score)  The number of participants in different genotype subgroups are listed in **Supplementary Table 2**. | | | | | | | | |
