# Supplementary material for: Deciphering the Impact of EPHA1‐AS1 Gene Polymorphism on Social Cognition Deficits in Parkinson's Disease
Source: CNS Neurosci Ther. 2026 Mar 27;32(4):e70801. doi: 10.1002/cns.70801 (PMC13140347; doi:10.1002/cns.70801)
Supplement: Supplementary file 5 — Table S5: Statistical power of comparisons between and within groups after controlling covariates. [file CNS-32-e70801-s006.docx]

| **Supplementary Table 5.** Statistical power of comparisons between and within groups after controlling covariates | | | | |
| --- | --- | --- | --- | --- |
|  | between PD and NCs^†^ | | within NCs^†^ | within PD^‡^ |
|  | aa+aA | AA |  |  |
| ***rs12703526*** | 0.709 | 0.986 | 0.987 | 0.691 |
| ***rs11771145*** | 0.991 | 0.620 | 0.987 | 0.691 |
| ***rs7805776*** | 0.970 | 0.844 | 0.987 | 0.691 |
| ***rs9640385*** | 0.850 | 0.966 | 0.987 | 0.691 |
| ***rs9640386*** | 0.925 | 0.931 | 0.987 | 0.691 |
| ***rs2966700*** | 0.972 | 0.833 | 0.987 | 0.691 |
| ***rs2949770*** | 0.649 | 0.990 | 0.987 | 0.691 |
| Abbreviations: NCs, normal controls; PD, Parkinson’s disease; a: minor allele, A: major allele  ^†^f=0.25, alpha=0.0125, covariates=4 (sex, age, education level, Mini-Mental State Examination score) under the ANCOVA model of G*power; ^‡^f=0.25, alpha=0.0125, covariates=6 (sex, age, education level, Mini-Mental State Examination score, levodopa equilivant daily dose, Hoehn-Yahr stage) under the ANCOVA model of G*power | | | | |
