# Supplementary material for: Deciphering the Impact of EPHA1‐AS1 Gene Polymorphism on Social Cognition Deficits in Parkinson's Disease
Source: CNS Neurosci Ther. 2026 Mar 27;32(4):e70801. doi: 10.1002/cns.70801 (PMC13140347; doi:10.1002/cns.70801)
Supplement: Supplementary file 6 — Table S6: Results of the moderation effect of rs2966700 on RMET positive subscore derived from the regression analysis. [file CNS-32-e70801-s001.docx]

| **Supplementary Table 6.** Results of the moderation effect of rs2966700 on RMET positive subscore derived from the regression analysis | | | | |
| --- | --- | --- | --- | --- |
| Variable | Coeff. | SE | t | *p* value |
| constant | 3.9352 | 0.9257 | 4.2510 | <0.0001* |
| X: rs2966700 | 0.4644 | 0.1692 | 2.7446 | 0.0063* |
| W: Group ^†^ | 0.2219 | 0.0655 | 3.3901 | 0.0008* |
| XW Interaction | -0.1083 | 0.0328 | -3.3016 | 0.0010* |
| sex | -0.5555 | 0.1594 | -3.4845 | 0.0005* |
| age | -0.0134 | 0.0049 | -2.7391 | 0.0064* |
| education level | 0.0639 | 0.0223 | 2.8713 | 0.0043* |
| MMSE score | -0.0398 | 0.0307 | -1.2972 | 0.1952 |
| Abbreviations: RMET, Reading the Mind in the Eyes Test; Coeff., coefficients; SE, standard error; MMSE, Mini-Mental State Examination, *p<0.0125  ^†^PD group is coded as 1 and normal controls is coded as 6. | | | | |
